# Identification of *CCNB2* as A Potential Non-Invasive Breast Cancer Biomarker in Peripheral Blood Mononuclear Cells Using The Systems Biology Approach

**DOI:** 10.22074/cellj.2021.7053

**Published:** 2021-08-29

**Authors:** Raheleh Moradpoor, Hakimeh Zali, Ahmad Gharebaghian, Mohammad Esmaeil Akbari, Soheila Ajdari, Mona Salimi

**Affiliations:** 1. Department of Basic Sciences, School of Allied Medical Sciences, Shahid Beheshti University of Medical Sciences, Tehran, Iran; 2. School of Advanced Technologies in Medicine, Shahid Beheshti University of Medical Sciences,Tehran, Iran; 3. Department of Hematology and Blood Banking, School of Allied Medical Sciences, Shahid Beheshti University of Medical Sciences, Tehran, Iran; 4. Cancer Research Center, Shahid Beheshti University of Medical Sciences, Tehran, Iran; 5. Department of Immunology, Pasteur Institute of Iran, Tehran, Iran; 6. Department of Physiology and Pharmacology, Pasteur Institute of Iran, Tehran, Iran

**Keywords:** Biomarker, Breast Cancer, Peripheral Blood Mononuclear Cell, Systems Biology

## Abstract

**Objective:**

Breast cancer (BC) still remains an imperative clinical issue, despite advances in the diagnosis, prognosis and treatment modalities of this malignancy. Hence, progress has been made to identify non-invasive, high sensitive and specific biomarkers. Since immune system affects development of breast cancer, peripheral blood mononuclear cells (PBMCs) -a subpopulation of immune cells- can be considered as a promising tool in the field of BC biomarker research. In the current study, we initially attempted to use concept of the present shared biomarkers in solid tumors and systemic immune profile and then evaluate correlation of these biomarkers to clinical use in cancer research.

**Materials and Methods:**

In this experimental study, available microarray gene expression datasets of BC as well as the related PBMCs were retrieved and downloaded from the Gene Expression Omnibus (GEO) database, followed by analysis using GEO2R along with affylmGUI, a R-based package, to obtain differentially expressed genes (DEGs). Signature genes from 20 types of cancer were also applied to validate DEGs. Quantitative reverse-transcription polymerase chain reaction (qRT-PCR) was carried out to assess mRNA level of CCNB2 in PBMC of the BC patients and healthy subjects.

**Results:**

DEGs analysis for the transcription profile of BC cells and PBMCs showed two shared targets, *CCNB2* and *PGK1*. Validation with systems biology using reweighted 20 types of cancer signature genes revealed that *CCNB2* is the only common target in BC and its related PBMCs, which was further validated by qRT-PCR implying a significant increase in the level of *CCNB2* in the BC patients.

**Conclusion:**

Results of this study demonstrated that PBMCs are affected by BC cells and *CCNB2* may be of value as a diagnostic biomarker for breast cancer. However, verification would require future detailed experimental plans.

## Introduction

One of the most common causes of cancer-related mortality in women throughout the world is breast cancer (BC) (1). Based on the statistics reported in 2012 by GLOBOCAN, despite rapid advances in effective and targeted therapy of BC, recurrence rate of this cancer is still high leading to reduced patients survival rate (2). Hence, timely screening is of paramount importance in the treatment and early diagnosis of BC patients. Imaging techniques have remarkably facilitated non-invasive approaches in early diagnosis; however, these techniques lose sensitivity when compared to the molecular biomarkers measured in blood. In this regard and considering cancer as a developing ecosystem rather than being merely a local disease, tracking its fingerprint in other tissues may also be of great value (3). For instance, determining prostate-specific antigen (PSA) levels in prostate cancer has provided a fast and effective screening modality (4). Considering the lack of such biomarkers for BC screening purposes in the clinical settings, investigations to find such markers would be of great value (5).

Interestingly, peripheral blood mononuclear cells (PBMCs) have been studied as a prognostic and diagnostic factor in cancer, due to their specific cellular and molecular features (6). PBMCs, as an important component of host immune cells, include lymphocytes, natural killer (NK) cells, dendritic cells and monocytes (7), which display a wide range of responses toward cancer cells (8). In other words, tumor cells secrete soluble factors causing immune cells to undergo various phenotypic and functional changes, which in turn, result in elimination, progression or invasion of cancer cells (9). Research has identified transcriptome profile of PBMCs as a valuable tool in distinguishing healthy individuals from cancer patients as well as recognizing tumor types including lung cancer, colorectal cancer, renal cancer, pancreatic cancer, melanoma and BC (10-12). Despite all the efforts, there is still a need to evaluate molecular profile of PBMCs to introduce the novel biomarkers associated with solid tumors (9). Moreover, PBMCs will be of clinical value, as blood sample collection and are more patient-friendly, less expensive, convenient and non-invasive rather than tumor tissue biopsy (13).

Systems biology is sought as an appropriate approach in discovering cancer related biomarkers (14). Systems biology approach consists of: i. Integration of omics data, ii. Reconstruction of molecular interaction networks and iii. Analysis of molecular pathways (9). It is worth mentioning that systems biology can contribute to the discovery of biomarkers based on the omics data, including genomics, transcriptomics, epigenomics, proteomics and metabolomics (15). Current approaches select potential targets solely based on differential expression of a panel of genes between healthy and diseased tissues. However, the obtained patterns of expression may not be validated in extensive cohort studies, as many genes are only transiently expressed following changes in the microenvironment of cancer cells. To avoid such inconsistencies, cancer systems biology is believed to provide more clear view of the dynamic nature of cancer (9).

To this end, the present study was aimed at integrating the available omics data in order to reconstruct and analyze the molecular interaction networks with the hope of identifying the shared targets between BC cells and the related PBMCs. The approach included identification of genes differentially expressed between BC tumor and the related PBMCs, compared to those of the healthy individuals using microarrays, followed by selection of common gene targets. These targets were then validated using previously reported cancer signature genes. Here we reported cyclin B2 (CCNB2) as the only shared protein target in BC tissues and the related PBMCs with a critical mechanistic role in the proliferation of BC.

## Materials and Methods

Systematic bioinformatics analysis and experimental design were used in this research.

### Selection of transcriptomics data set

**Fig.1:**
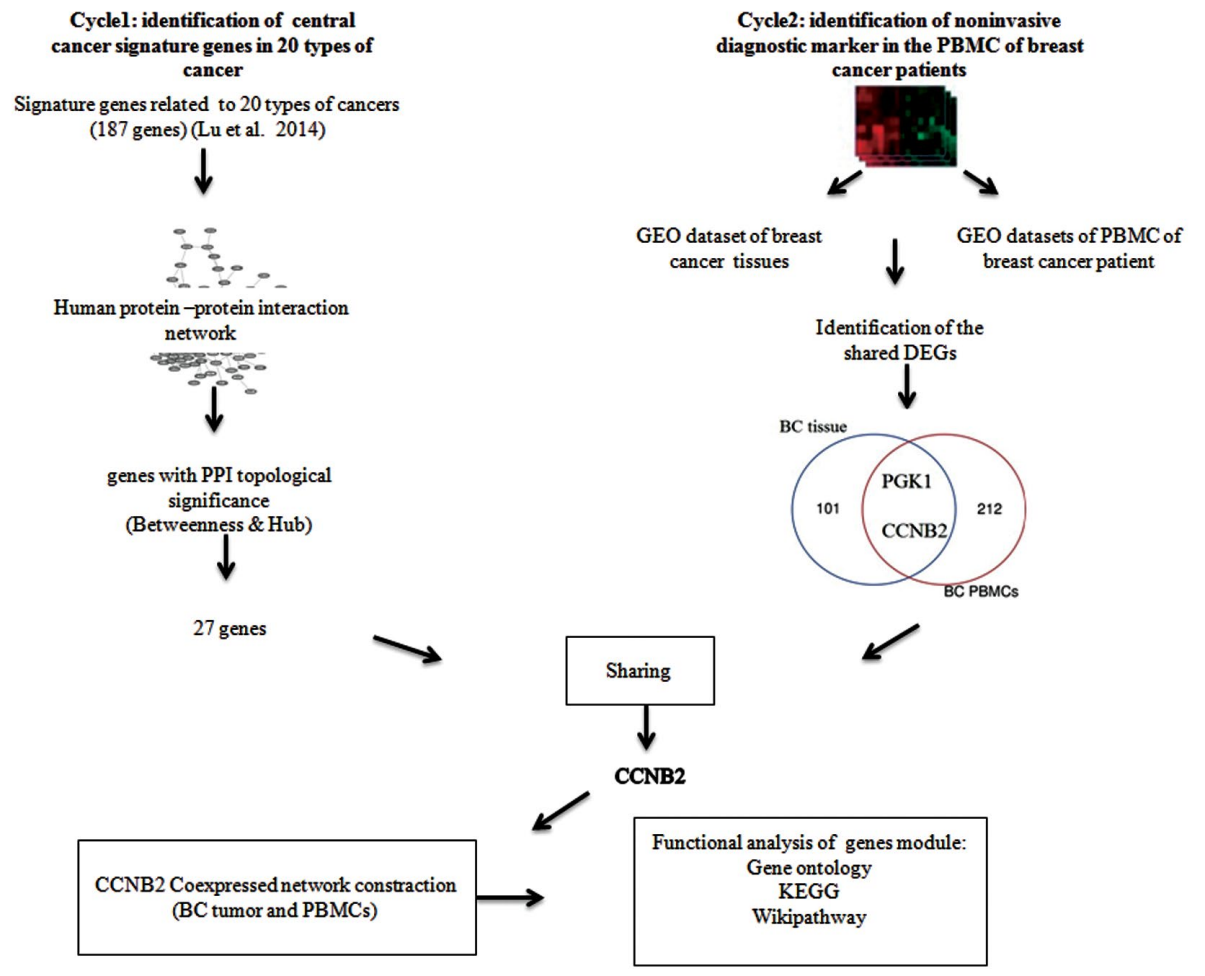
Strategy of the shared target discovery in cancer ecosystem. Two-cycle procedures were applied in this study. The first cycle reveals the identification of central cancer genes and the second one demonstrates the valuable non-invasive cancer diagnostic markers shared with the central cancer signature genes. PPI; Protein-protein interaction network, BC; Breast cancer, DEGs; Differentially expressed genes, PBMCs; Peripheral blood mononuclear cells, and GEO; Gene expression omnibus.

As presented in Figure 1, our workflow revealed a shared gene between BC tissue and its related PBMCs. Cancer signature gene sets, publically available by Meng et al. (16) and Lu et al. (17) were applied to construct the interaction network using the STRING web tool (version 11.0) (http://www.string.embl.de/). Cytoscape software (version 3.5.1) was used for network visualization. Analysis of topological properties of the network, including connectivity degree of each node and betweenness centrality, was calculated using Network Analyzer plugin in the Cytoscape. 

We retrieved and downloaded microarray expression datasets from the Gene Expression Omnibus (GEO) database for PBMC and tumor sample of the BC patients [GSE21422(26), GSE10797(27), GSE3744(28), GSE27567(29)].

A web tool, GEO2R (http://www.ncbi.nlm.nih.gov/ geo/geo2r/) along with affylmGUI, a R-based package, were utilized for the analysis of GEO data sets to obtain differentially expressed genes (DEGs). Log fold changes of more than 0.5 and adjusted P<0.05 were considered significant.

Co-expression network of the signature genes was constructed with the ARACNE tool. Pathway and gene ontology (GO) enrichment analysis of the genes, expressed differentially in the co-expression network, was carried out with Enrichr web tool (https://amp.pharm. mssm.edu/Enrichr/). 

Survival rates were predicted using Kaplan-Meier by the web tool accessible at http://www.compbio.iupui. edu/proggene (18). This tool performs validation and prognostic analysis of the available gene expression datasets. Hazard ratio (HR) was used to predict impact of genes on the survival rate. A P<0.05 was considered as statistically significant.

### Quantitative measurement of *CCNB2* mRNA level

PBMC samples of the patients, histologically diagnosed as metastatic and non-metastatic BCs (24 cases of BC), were subjected to quantitative reverse-transcription polymerase chain reaction (qRT-PCR) analysis by Rotor-Gene Q (Qiagen, Germany) to determine *CCNB2* mRNA levels. Clinicopathological characteristics of the patients were obtained from their medical records. All samples were collected with informed consent from patients and the current study protocol was established by the Human Ethics Research Committee of Shahid Beheshti University of Iran (IR.SBMU.RETECH.REC.1398.688). For qRT-PCR, following the extraction of RNA using Trizol (Invitrogen, USA), RNA was reverse-transcribed by the Superscript First-Strand Synthesis System (ThermoFisher Scientific, USA). Primers for *CCNB2* and *GAPDH*, as a housekeeping gene, were designed by AlleleID6 software (PREMIER Biosoft International, USA) and checked against the GenBank database to ensure that no similarities remained with other known human DNA sequences (Table S1, See Supplementary Online Information at www.celljournal.org). The ΔΔCt method was used to calculate the comparative gene expression levels. 

## Results

### Common target discovery in breast tumor and the related PBMCs

**Fig.2:**
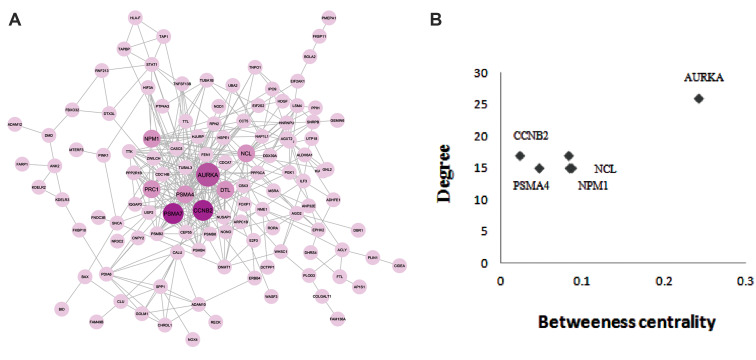
One hundred and eighty seven cancer signature genes in twenty types of cancer were used to construct the PPI network. **A.** PPI map of the cancer signature genes involving in 320 nodes and 129 interactions, major hubs are highlighted. **B.** Analysis of network topology, including degree and betweeness centrality indicates major PPI hubs. PPI; Protein-protein interaction.

In the experimental study, we firstly attempted to use concept of the present shared biomarkers in the solid tumors and systemic immune profile and then find potential correlation of these biomarkers to clinical use in cancer research. To provide a prediction with high success rate, 187 cancer signature genes, including 117 up-regulated and 70 down-regulated ones, were used in all twenty types of cancer (Supplementary File 1, See Supplementary Online Information at www.celljournal.org). Using STRING web-tool, a total of 320 interactions and 129 genes were extracted to construct a PPI network (Fig .2). Genes in the network were reweighted relying on their topological features, followed by calculating the topological properties of network, including connectivity degree of each node and betweenness centrality. Finally, top 20% of the genes with the highest betweenness value were selected. Degree distribution of the network and goodness of fit with a power-law distribution were determined using R-square (Table S2, See Supplementary Online Information at www.celljournal.org). High R-square indicates the existence of fat tail in the degree distribution, implying that some genes can be hubs (19). Knowing this, the top six nodes with degrees ≥14.29 (*AURKA, PSMA7, CCNB2, NCL, NPM1 *and* PSMA4*) were selected and considered as hub genes with the ability to interact with a wide range of proteins (20). Noteworthy, hub genes play a decisive role in cancer development and they can be utilized as diagnostic, prognostic and therapeutic biomarkers (21). As the next step, the obtained hub genes were combined with genes possessing the highest betweenness centrality, determined initially, in order to construct a conclusive set of central cancer signature genes (Table S3, See Supplementary Online Information at www.celljournal.org). 

On the other hand, in a second pipeline, DEGs were identified from both GEO BC tissue datasets and PBMCs, as mentioned in the materials and methods section. Following the comparison of DEGs in datasets of the both groups, common DEGs were identified. The common dysregulated genes are shown in Table 1. As indicated in Table 1, *CCNB2* and *PGK1* are the genes overexpressed in both PBMCs and breast tumors. Interestingly, as the two genes drive cancer progression, they can be used as BC signature genes (22, 23). As final step, a search into the conclusive set of central cancer signature genes revealed *CCNB2* as the only common gene overexpressed in the all datasets. Thus, *CCNB2* was introduced as a potential diagnostic marker for BC screening purposes, due to the significant fluctuations in its expression level in the cancerous tissue versus the related PBMCs, in addition to playing an essential role in the sense of topologic centrality in protein-protein interaction network.

**Table 1:** DEGs of breast cancer tumor and the corresponding PBMC transcriptome data indicated in each GO term


Samples	DEGS	Shared genes between BC tumor and PBMC	Shared genes with Betweenness+Hub	Genes	Consensus gene between BC tumor and PBMC with Betweenness+Hub

BC tumor	103	*CCNB2, PGK1*	15	*AURKA, CCT5, NME1, PSMA7, SNCA, EIF2S2, SPP1, STAT1, DTX3L, PSMA4, KDELR3, FBXO32, EPRS, ALDH6A1, CCNB2*	*CCNB2*
BC PBMC	213	*CCNB2, PGK1*	2	*ACLY, CCNB2*	*CCNB2*


DEGs; Differentially expressed genes, BC; Breast cancer, and PBMCs; Peripheral blood mononuclear cells.

### Identification of *CCNB2* co-expressed genes module

**Fig.3:**
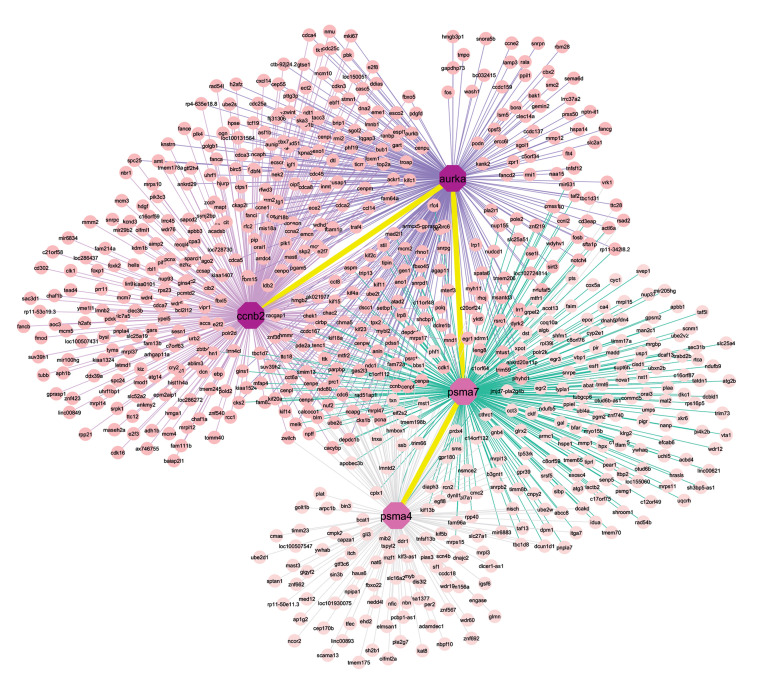
Co-expression network of four major hubs in breast cancer tissue; purple nodes are hub genes of the network. Yellow lines indicate interaction between the hub genes.

ARACNE is an established tool to identify modules of biologically related genes (24). On the other hand, co-expression network analysis is an approach to recognize genes with similar functions and association with the disease under study (25). In the current study, we carried out ARACNE analysis on the preprocessed normalized data of BC tumor tissue and the related PBMCs to identify *CCNB2* co-expressed gene modules (Supplementary File 2, See Supplementary Online Information at www. celljournal.org). The selected gene modules were then subjected to further analysis to construct a data set network (Fig .3). Overall, the data set consisted of 361 nodes and 360 edges in the tumor tissue of BC along with five nodes and four edges in the related PBMCs of BC. 

### Gene ontology analysis of the co-expressed gene module of *CCNB2*

Enrichr was utilized to perform GO analysis in order to explore the significant molecular functions (MF), cellular compartments (CC) and biological processes (BP) associated with the genes presented in the co-expression gene module of *CCNB2* (Table 2). Enrichr database uses Fisher exact test to compare the input gene list with the gene libraries (26). An adjusted P value of less than 0.05 and maximum numbers of overlapped genes in each ontology analysis were considered as a threshold. The number of 211 BP, 47 MFs and 48 cellular components GO terms were recognized to be related to the co-expression genes module of *CCNB2*. Overall, BP analysis revealed that multiple co-expressed genes were predominantly involved in cell cycle regulation and DNA replication processes. As an example, we can address DNA metabolic and replication (GO: 0006260; GO: 0006259), microtubule cytoskeleton organization implicated in mitosis (GO: 1902850), mitotic cytokinesis (GO: 0000281), mitotic spindle organization (GO: 0007052), regulation of cell cycle processes (GO: 0010564) and G1/S transition of mitotic cell cycle (GO: 0000082). Moreover, associated CC were as follows: centromeric region (GO: 0000775), mitotic spindle (GO: 0072686), microtubule cytoskeleton (GO: 0015630) and condensed nuclear chromosome kinetochore (GO: 0000778).

As a further step, GO enrichment analysis of *CCNB2* co-expressed genes module was carried out in cancerous PBMCs. This revealed similar results in both BP and CC. 

**Table 2:** GO enrichment analysis (biological process) of *CCNB2* co-expression network. Hub genes indicated in each GO term


Ontology-ID	Ontology name	Adjusted P value	Overlap	Hub genes	Other genes in the network

0044772	Mitotic cell cycle phase transition	4.21E-18	33/222	*AURKA CCNB2 *	*MCM7,MCM10,FOXM1,AURKA,CCNB2,CDC45,ORC1,RCC1,NEK2,PLK4,CDT1,UBE2C,TUBB,PLK1,CDC6,MASTL,CDC25C,CCNA2, MELK,DBF4,UBE2S,CCNE1,MCM3,CDK1, MCM4,MCM5,MCM6,MCM2,CDC25A, TACC3,CCNB1,RANBP1,CDKN3*
1902850	Microtubule cytoskeleton organization involved in mitosis	7.28E-15	16/45	*AURKA*	*STIL,TTK,KIF11,AURKB,NDC80,AURKA,CENPE,CCNB1,ESPL1,KIF4A,NUF2,STMN1TACC3,RCC1,SPC25,NUSAP1*
0007052	Mitotic spindle organization	9.4E-15	19/75	*AURKA*	*STIL,TTK,KIF23,KIF11,AURKB,NDC80,AURKA,TPX2,CENPE,CCNB1,PRC1,KIFC1NUF2,STMN1,RCC1,BIRC5,MYBL2,SPC25KIF4A*
0010564	Regulation of cell cycle process	4.5E-11	17/91	*AURKA*	*TIPIN,RMI2,PLK1,FOXM1,MKI67,CDC25CAURKB,AURKA,RACGAP1,PRC1,RCC1,NEK2FBXO5,KIF20A,ECT2,KNSTRN,PIK3C3*


### Pathway analysis of the co-expressed genes module of *CCNB2*

The genes co-expressed in the *CCNB2* network were further investigated for their presence in the curated KEGG and Wiki Pathways collections. KEGG and Wiki Pathways collections are public repository of the curated and dynamic models of BP (27). A total of 30 human pathways were identified in which at least one of the genes from the co-expressed network of *CCNB2* was included. In addition to the identified *CCNB2* in eleven pathways, a second gene -*AURKA*- included in the hub cancer signature genes, was also identified.

Furthermore, pathways including either a maximum number of 30 genes or the genes from cancer signature were recognized. These pathways represented highly associated BP involving in *CCNB2*. Among the identified pathways, two pathways including cell cycle (KEGG) and retinoblastoma (*Rb*) gene in cancer (WP2446) have the maximum number of genes (30 genes), in which 18 genes were shared. 

Studies revealed role of *Rb* gene in the signaling pathways involved in cancer progression during embryonic development and oocyte maturation. In other words, functional Rb is required in a subset of malignancies to maintain proliferation and prevent apoptosis of cancer cells (28). Several studies identified that CDK-RB-E2F pathway was vital for controlling cell proliferation, while others assigned additional roles for this pathway in angiogenesis, metastasis and cancer progression, particularly in BC (29). In addition to Rb, cell cycle checkpoints were crucial in cancer progression, so that dysregulation of the checkpoints led to an uncontrolled proliferation (30). 

Furthermore, among the identified pathways, 2 hub genes (*CCNB2* and *AURKA*) were shared in the oocyte meiosis and progesterone-mediated oocyte maturation pathways. It is worth mentioning that pathways including p53 signaling, cellular senescence, human T-cell leukemia virus 1 infection, cell cycle (WP179), DNA replication (WP466), miRNA regulation of DNA damage response (WP1530) and DNA damage response (WP707) only shared *CCNB2* gene from the hub genes, while *AURKA* was only detected in the gastric cancer network 1 (WP2361) pathway. These observations are in line with the previous studies confirming the role of *CCNB2* and *AURKA* in cancer cell progression (31). Overall, results of this study revealed several critical signaling pathways and associated co-expressed *CCNB2* gene, which needs to be experimentally validated.

### Prognostic impact of *CCNB2* in breast cancer patients

**Fig.4:**
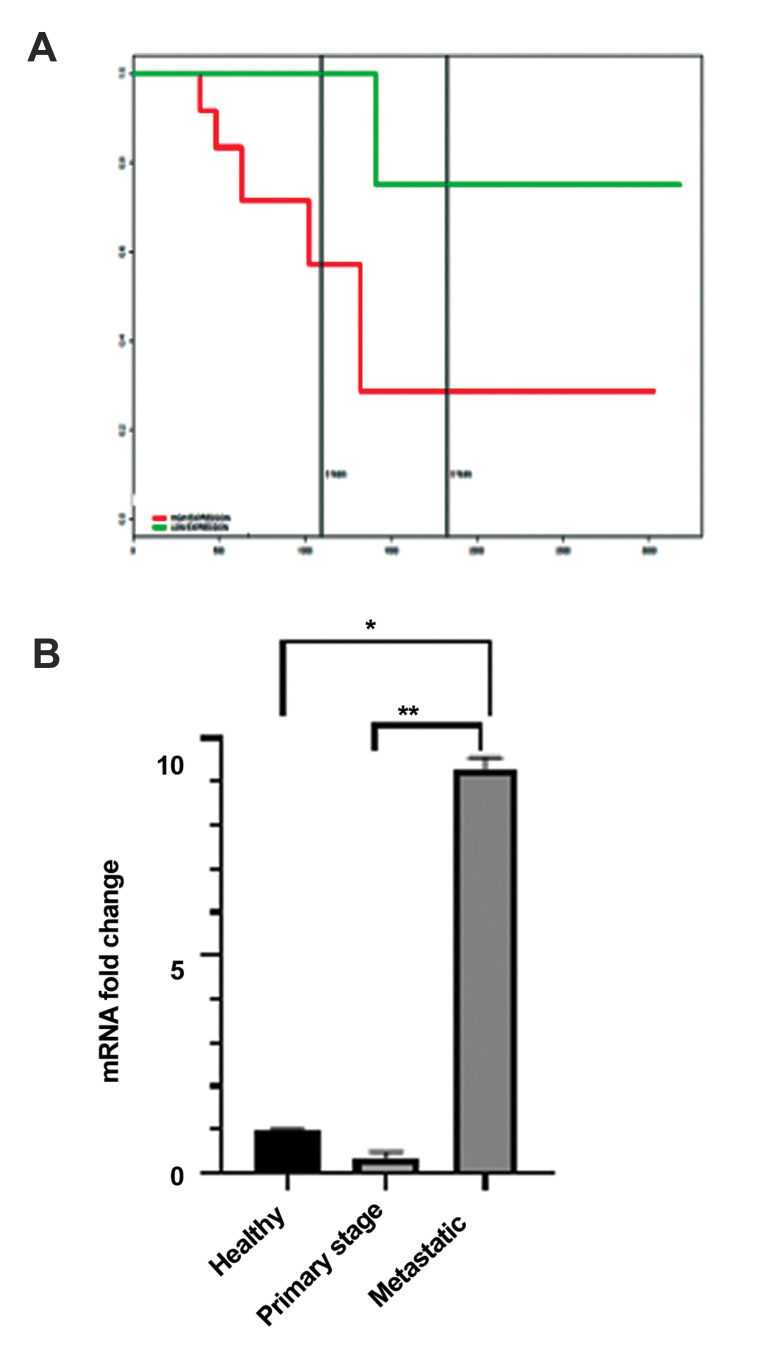
*CCNB2* gene expression analysis in PBMCs from the healthy individuals and patients with primary and metastatic stages of BCs. **A.** Kaplan-Meier plot using *CCNB2* gene shows survival rate of the patients in the study. **B.** mRNA quantification of *CCNB2* level in PBMCs of healthy individuals, primary and metastatic stages of breast cancer patients. Kruskal-Wallis test was performed. PBMCs; Peripheral blood mononuclear cells, BC; Breast cancer, * ; P<0.05, and **; P<0.01.

At the time of diagnosis, prognostic markers have potential of supplying information on clinical consequences, independent of therapy. In cancer, prognostic biomarkers indicate growth, invasion and metastatic features of cancer (32). Herein, we attempted to evaluate prognostic impact of *CCNB2* as a proliferation marker on the survival rate of BC patients. To this end, survival rates were measured using the Kaplan-Meier method and the findings showed a specific death as a result of BC (33). Survival analysis was performed on the BC dataset (GSE6130) for two-time points (3 and 5 years). As depicted in Figure 4A, over-expression of *CCNB2* resulted in low survival rate in the both time-points A B (HR=7.07, P=0.01). These findings indicated that *CCNB2* has a significant role in cancer progression.

### Transcriptional expression of *CCNB2* gene in the PBMC of breast cancer patients

In order to make the study more plausible, PBMCs were obtained from the both normal healthy individuals and patients with primary and metastatic stages of BC and then subjected to *CCNB2* gene expression analysis. Expression level of *CCNB2* was found to be higher in the metastatic patients (24.8 fold) than patients with primary stage (Fig .4B). Moreover, expression level of this gene in PBMCs of healthy subjects was 2.6 fold higher than PBMC of the patients in the primary stages. 

## Discussion

Gene expression profiling and the subsequent topological characterization of protein-protein interaction networks are among the central strategies in biomarker discovery (34). Since biological function of proteins depends on their interaction with other partners, each gene possesses its own topological characters. In this study, our aim was to identify proteins with a diagnostic potential in PBMCs of BC patients by taking advantage of insights from the analysis of protein-protein interaction networks. To achieve this, a search to find common markers between BC tumors and the corresponding PBMCs was initiated. To start with robust data, a total of 187 commonly dysregulated genes in 20 types of cancer were used as previously reported, owing to their high chance of carcinogenesis (16). These dysregulated genes were used in the construction of protein-protein interaction networks. Of these, a total of 27 candidate genes were selected according to the topological significance of the PPI network consisting of betweenness and a high connectivity degree. In analogy to the well-known network hubs, betweenness was chosen to measure complementary properties of node importance (35). These 27 candidate genes were named as central cancer genes with a role in cancer progression. Next, common genes between tumor and PBMC of the BC patients were matched to the central cancer genes. *CCNB2* was the only gene with consistent changes in the tumor and the associated PBMCs as well as the other cancer types. It was also concluded that *CCNB2* may be a significant DEG between breast tumor and the associated PBMCs corroborating our topological analysis and showing the highest degree value for *CCNB2* among the human protein-protein interaction networks in BC tissues. It is worthy to mention that *CCNB2* plays an essential role in cell proliferation and division based on our literature survey. Moreover, *CCNB2* is considered to be a potential diagnostic as well as progression biomarker (36). 

Functional analysis of the genes module of *CCNB2* co-expressed network revealed an association of these genes with the BP, such as DNA replication and cell cycle regulation. These results are in agreement with the previous studies, regarding the function of *CCNB2*. *CCNB2* gene, a member of the B-type cyclin family, is involved in cell proliferation and tumor progression (36- 38). It is well-established that cyclins along with cyclin-dependent kinases (cdks) are two crucial regulatory molecules involved in the progress of cells through cell cycle (36, 37). In particular, cyclin B is crucial for mitosis and DNA synthesis stages of the cell cycle. It is highly expressed through transition from G2 to M phases and degraded during the anaphase. It also contributes to the G2-M transition via modulating CDC2 kinase, while its inhibition leads to cell cycle arrest. Finally, dysregulated expression of the cell cycle–related proteins is closely connected to tumorogenesis and cancer progression (38).

Serum circulating *CCNB2* mRNA may have clinical applications in tracking of metastasis as well as prognosis of BC as a biomarker (36, 39). Interestingly, no statistically significant difference was reported for CCNB2 protein level in terms of patient age, tumor grade, tumor size, ER/ PR/HER2 status, stage and axillary lymph node status (36). In accordance with previous studies, our survival analysis highlighted CCNB2 as a promising prognostic marker in BC patients with an overall shorter survival rate (23, 36). 

In addition to *CCNB2*, a second module, *AURKA*, in BC tissues, was found to be highly connected to *CCNB2* module. Indeed, CCNB2 acts as a critical node bridging two modules to each other. Interestingly, our findings displayed a positive correlation between hub characteristic and survival rate, despite *CCNB2* lacking a high betweenness in cancer signature genes network. Accumulating evidences suggest that CCNB2 and p53 tumor-suppressor proteins function antagonistically in regulating aurora-A-mediated centrosome separation and correcting chromosome segregation in the late G2 by provoking the phosphorylation of aurora A (AURKA). AURKA, a serine/threonine kinase, is a vital oncogene in epithelial–mesenchymal transition, cancer stem cell development and distant metastasis (37). AURKA is regarded as a standard proliferation marker in the Oncotype DX test for assessment of the risk of distant recurrence of BC (40).

Given the protein-protein interaction, functional and survival analyses, we propose CCNB2 and AURKA hub modules as potential drivers of proliferation and metastasis in breast cancer cells. Taking into account that CCNB2 was the only shared protein in the PBMCs of BC and knowing that mRNA expression level of *CCNB2* was elevated in PBMCs of the metastatic BC patients, we introduce it as a diagnostic candidate biomarker in the progression of BC patients using blood sampling. 

## Conclusion

We conclude that PBMCs can effectively reflect tumor behavior, suggesting the important role of PBMCs transcriptome in tumor development and its subsequent application for cancer diagnosis. Considering the up-regulation of *CCNB2* in BC tissues as well as its related PBMCs compared to healthy tissues, it is thus suggested as a novel diagnostic approach of solid tumors. 

## Supplementary PDF


