# Supplementary material for: Identification of CCNB2 as A Potential Non-Invasive Breast Cancer Biomarker in Peripheral Blood Mononuclear Cells Using The Systems Biology Approach
Source: Cell J. 2021 Aug 29;23(4):406–13. doi: 10.22074/cellj.2021.7053 (PMC8405074; doi:10.22074/cellj.2021.7053)
Supplement: Supplementary file 1 [file Cell-J-23-406-s01.pdf]

www.celljournal.org

Supplementary Information for

Identification of *CCNB2* as A Potential Non-Invasive Breast Cancer Biomarker in Peripheral Blood Mononuclear Cells Using The Systems Biology Approach

Raheleh Moradpoor, Ph.D.[1], Hakimeh Zali, Ph.D.[2], Ahmad Gharebaghian, Ph.D.[3], Mohammad Esmaeil Akbari, M.D.[4], Soheila Ajdari, Ph.D.[5], Mona Salimi, Ph.D.[6]*

1. Department of Basic Sciences, School of Allied Medical Sciences, Shahid Beheshti University of Medical Sciences, Tehran, Iran
2. School of Advanced Technologies in Medicine, Shahid Beheshti University of Medical sciences,Tehran, Iran
3. Department of Hematology and Blood Banking, School of Allied Medical Sciences, Shahid Beheshti University of Medical Sciences, Tehran, Iran
4. Cancer Research Center, Shahid Beheshti University of Medical Sciences, Tehran, Iran
5. Department of Immunology, Pasteur institute of Iran, Tehran, Iran
6. Department of Physiology and Pharmacology, Pasteur Institute of Iran, Tehran, Iran

**Corresponding Address: P.O.Box: 13164, Department of Physiology and Pharmacology, Pasteur Institute of Iran, Tehran, Iran*
Email: salimi_mona@yahoo.com

Table S1: qPCR primers for assessing *CCNB2* mRNA expression level using qRT-PCR

Primers	Primer sequence (5′- 3′)
CCNB2	F: TTACTGCTCTGCTCTTGGCTTC
	R: TCTCGGATTTGGGAACTGGTATAAG
GAPDH	F: ACATCAAGAAGGTGGTGAAGCAG
	R: GCGTCAAAGGTGGAGGAGTG

QRT-PCR; Quantitative reverse-transcription polymerase chain reaction.

Table S2: Power-law behavior of the node distribution of 187 cancer signature genes network, (10.9 KB docx)

Power-law distribution	Correlation coefficient of power-law fit (R)	R-squared of power-law fit*	R-squared of best line- fit
y ≈ 39.166x -1.028	0.961	0.82	0.413

*; R-Squared is computed on logarithmized values.

Table S3: Central cancer signatures genes are conclusive set of Hub genes combined with betweenness centrality (13.9 KB docx)

No.	Betweenness centrality	Hub	Betweenness centrality + Hub
1	*AURKA*	*AURKA*	*AURKA*
2	*PDIA6*	*PSMA7*	*PDIA6*
3	*CCT5*	*CCNB2*	*CCT5*
4	*NME1*	*NCL*	*NME1*
5	*PSMB8*	*NPM1*	*PSMB8*
6	*ACLY*	*PSMA4*	*ACLY*
7	*NCL*		*NCL*
8	*NPM1*		NPM1
9	*PSMA7*		*PSMA7*
10	*PSMB2*		*PSMB2*
11	*HNRNPK*		*HNRNPK*
12	*TUBA1B*		*TUBA1B*
13	*FKBP10*		FKBP10
14	*SNCA*		*SNCA*
15	*EIF2S2*		*EIF2S2*
16	*EIF2AK1*		*EIF2AK1*
17	*SPP1*		*SPP1*
18	*STAT1*		STAT1
19	*DTX3L*		*DTX3L*
20	*PSMA4*		*PSMA4*
21	*KDELR3*		*KDELR3*
22	*DNMT1*		*DNMT1*
23	*FBXO32*		*FBXO32*
24	*EPRS*		*EPRS*
25	*ALDH6A1*		*ALDH6A1*
26	*PLOD3*		PLOD3
			CCNB2

The top 20% of genes with highest betweenness value were selected. Conclusive set of Hub genes combination with betweenness centrality was named central cancer signatures genes.
